# Association of autoimmune pancreatitis with Raghib syndrome

**DOI:** 10.1002/ccr3.8194

**Published:** 2023-12-18

**Authors:** Azizollah Yousefi, Shima Salehi, Mohammad Radgoodarzi, Asma Javid

**Affiliations:** ^1^ Department of Pediatrics Hazrat‐e‐Rasool General Hospital Iran University of Medical Sciences Tehran Iran; ^2^ Department of Pediatrics Hazrate Ali Asghar Children Hospital Iran University of Medical Sciences Tehran Iran; ^3^ Department of Pediatric Firouzabadi Clinical Research Development Unit Iran University of Medical Sciences Tehran Iran

**Keywords:** autoimmune pancreatitis, echocardiography, IgG4‐related disease, left superior vena cava, Rhaghib syndrome

## Abstract

**Key Clinical Message:**

Autoimmune pancreatitis (AIP) is a form of chronic pancreatitis scarcely found in children. Raghib syndrome is a rare congenital heart defect known as persistent left superior vena cava (LSVC) draining into the left atrium. Total signs of Raghib syndrome in AIP case accompanied by an IgG4‐related disease were described.

**Abstract:**

Autoimmune pancreatitis (AIP) is a form of chronic pancreatitis scarcely found in children. Raghib syndrome is a rare congenital heart defect known as persistent left superior vena cava (LSVC) draining into the left atrium. Here, we describe Raghib syndrome in AIP case accompanied by an IgG4‐related disease (AIP/IgG4RD). A 13‐year‐old boy presented with a 3‐month history of fever and abdominal pain. The laboratory findings showed SGOT and SGPT, ALP was increased, while amylase and γ‐GT were normal. Immunoglobulins were normal, except for IgG. Endosonography, spiral CT of the abdomen, and cholangiopancreatography showed an enlargement of the pancreas. Contrast echocardiography discovered opacification of the coronary sinus and left atrium. Transesophageal echocardiography for LSVC revealed a dilatation in the coronary sinus, indicating persistent LSVC. Following the injection of agitated saline into the left antecubital vein, bubbles entered both left and right atria in LSVC. It is reasonable to exclude some of these rare disorders as Raghib syndrome, in cases that will be started on medications like corticosteroids, which increases the susceptibility to thromboembolic events.

## 1 INTRODUCTION

Autoimmune pancreatitis (AIP) is a recently characterized form of chronic pancreatitis, which occurs scarcely. Clinical, histologic, and morphologic characteristics of AIP comprise lymphoplasmacytic sclerosing pancreatitis, chronic sclerosing pancreatitis, nonalcoholic duct‐destructive chronic pancreatitis, and inflammatory pseudotumor.^1^ It has been scarcely described in children.^2^ From the clinical viewpoint, patients may complain of abdominal pain, jaundice, weight loss and may present obstructive patterns in liver function tests (LFT). There is usually diffuse enlargement of the pancreas in imaging with diffusely or segmentally narrowed pancreatic ducts.^3^

A new clinicopathologic concept of IgG4‐related systemic disease (ISD) has been proposed by Kamisawa et al.^4^ In fact, ISD is defined as a syndrome characterized by elevated serum IgG4 levels, prominent lymphoplasmacytic infiltrate with increased IgG4^+^ plasma cells, dense sclerosis storiform fibrosis, and obliterative phlebitis. The fibrosis correlated with ISD may corrupt and even partially kill the affected organ; however, the inflammatory process typically responds to corticosteroid therapy.^3^, ^5^

The left superior vena cava (LSVC) is the most prevalent congenital malformation of the thoracic venous system, which is found in 0.3%–0.5% of the general population and with an incidence rate of up to 10% (10 folds higher) in patients suffering from congenital heart malformation.^6^, ^7^, ^8^ Notably, the LSVC itself causes no hemodynamic trouble and is often diagnosed incidentally.^9^ There is an increased risk of paradoxical embolism with LSVC due to its association with interatrial septal defect, unroofed coronary sinus, or direct communication to the left atrium.^7^

It drains into the right atrium via an enlarged coronary sinus in approximately 90% of patients. On rare occasions, the persistent left SVC directly enters the left atrium, creates a right to left shunt, resulting in partial anomalous systemic venous return.^10^ The standard method for the diagnosis is echocardiography, either transthoracic or transoesophageal. The most frequent echocardiographic feature of persistent LSVC (PSLVC) is a dilated coronary sinus and should hint the echocardiographer to look for a PLSVC. The diagnosis is then confirmed by contrast echocardiography. After excited saline injection into a left‐sided brachial vein, bubble contrast seems in the coronary sinus before appearing in the right atrium and ventricle. Raghib syndrome is a sporadic congenital heart defect characterized by persistent left superior vena cava (LSVC) draining into the left atrium and the absence of the coronary sinus.^10^ Here, we reported an uncommon finding of Raghib syndrome in a case with AIP accompanied by an IgG4‐related disease (AIP/IgG4RD).

## 2 CASE PRESENTATION

Our patient is a 13‐year‐old boy who presented with a 3‐month history of fever, anorexia, mild icterus, pale conjunctiva, myalgia, arthralgia, weight‐bearing pain, headache, epigastric and right upper quadrant pain. The abdomen was soft without tenderness or organomegaly. In physical examination, there was no detectable lymphadenopathy. He had a mild fever (37.8°C) and complained about a 5‐kg loss of weight during this period.

Laboratory findings included normocytic normochromic anemia, mild leukocytosis, increased erythrocyte sedimentation rate, normal prothrombin and partial thromboplastin time (PT and PTT), serum albumin and total protein, increased SGOT (49 IU/L) and SGPT (58 IU/L), alkaline phosphatase (ALP) (614 IU/L). Amylase (35 IU/L), lipase (20.27 IU/L), Gamma‐Glutamyl Transferase (γ‐GT) (48 U/L) all were in the normal range. Viral hepatitis markers, Wright and Coombs wright tests were negative. Rheumatoid factor and complement protein levels (C3, C4, and CH50) and anti‐LKM Ab, ANA (0.7), anti‐dsDNA (7 IU/mL), and alpha1 antitrypsin levels were in the normal range. Moreover, anti‐MPO antibody (P‐ANCA:3), PR3 antibody (C‐ANCA:2), and anti‐CCP (4.5 AU/mL) were negative. Anti‐CEA and CA19‐9 antibody levels were within normal limits.

Immunoglobulins (IgA, IgG, and IgM) levels were normal, except for IgG (188 IU/mL). Sterile pyuria (WBC: 40–45 and RBC: 2–3) was also detected. Abdominal sonography revealed the enlargement of the left liver lobe, pancreas, and lymph nodes in the mesenteric and paraaortic areas. It increased corticomedullary differentiation in both kidneys (Head: 30 mm, body: 10 mm, and tail: 12 mm). Dimercaptosuccinic acid (DMSA) showed a scar in the lower portion of the right kidney. The whole‐body scan was normal. Endosonography and spiral CT of the abdomen showed the prominence of head of pancreas and periceliac artery soft tissue infiltration, and enlarged kidneys. MR Cholangiopancreatography revealed an enlargement of the pancreas, standard caliber and length of common bile duct, and cystic duct, but a narrowed pancreatic duct with non‐homogenous internal structure. In addition, liver biopsy showed chronic hepatitis. Bone marrow aspiration also revealed erythroid hyperplasia and dyserythropoietic changes.

A thorough history was taken to assess probable cardiac complications of an inflammatory process, and the physical examination was followed by performing electrocardiography and echocardiography. Transthoracic echocardiography revealed mild mitral valve prolapse without verrucae and mild MR. He was also detected to have unexplained grossly dilated coronary sinus (CS). Since we must follow the existence of persistent LSVC in every person with dilated coronary sinus, it was observed as a retrograde flow from the upper neck toward coronary sinus in suprasternal view echocardiography. Subsequently, contrast echocardiography from both arms discovered opacification of the CS and left atrium before the right atrium, which raised the suspicion of an unroofed CS. To confirm this, transesophageal echocardiography (TEE) was performed. TEE with contrast revealed both RSVS and LSVC. Right SVC drainage was normal. Injecting agitated saline into the left antecubital vein, LSVC showed bubble entrance into the left and right atria.

## 3 DISCUSSION

Autoimmune pancreatitis (AIP) is a rare and newly recognized form of chronic pancreatitis, which can be misleadingly diagnosed as pancreatic cancer.^11^ This condition was first described in adults and is rarely reported in children.^3^ AIP is histologically characterized by diffuse lymphoplasmacytic infiltration, obliterative phlebitis, and interstitial fibrosis.^12^ Numerous affected patients have hypergammaglobulinemia and increased serum levels of IgG, particularly IgG4.^13^

Recently, Mayo Clinic introduced several criteria for diagnosing AIP, summarized by the mnemonic HI‐SORt, these criteria include five cardinal features of AIP in histology, imaging, serology, other organ involvements, and response to corticosteroid therapy.^14^, ^15^

AIP is infrequently associated with other autoimmune disorders such as Sjogren syndrome, idiopathic retroperitoneal fibrosis, and inflammatory bowel disease (IBD). Extrapancreatic manifestations include sclerosing cholangitis, renal mass or nephritis, retroperitoneal fibrosis, and submandibular masses.^16^

Raghib syndrome is an uncommon congenital heart defect described by persistent left superior vena cava (LSVC) draining into the left atrium and the absence of the coronary sinus.^17^, ^18^

To the best of our knowledge, the occurrence of Raghib syndrome or unroofed LSVC in a case with AIP has not been reported yet. We detected a dilated coronary sinus in echocardiography guided to find the PLSVC. In our patient, PLSVC was confirmed to have drainage to the left atrium as well.

Moreover, PLSVC is asymptomatic, and an incidental finding is often discovered incidentally during cardiovascular imaging or surgery, left subclavian vein cannulation, or device implantation.^19^

In this case, an echocardiographic examination revealed dilated coronary sinus that raised the suspicion of LSVC. Therefore, simple agitated saline bolus injections into the left antecubital veins (contrast echocardiography) were performed. Scanning healthy individuals in four‐chamber view reveals the saline contrast, administered thorough left upper extremity vein, first in the right atrium. In this regard, the contrast will first appear in the dilated coronary sinus then in the left atrium if there is PSVC drainage to the LA.

In a retrospective study, the patients who underwent either pacemaker or ICD implantation PLSVC were estimated at 0.41% of the general population, emphasizing critical clinical implications and difficulties during central venous access, cardiothoracic surgeries, and pacemaker implantation. When the left upper extremity is used for accessing, serious complications such as arrhythmia, tamponade, and coronary sinus thrombosis may occur due to manipulating the catheter in the CS.^20^

Furthermore, a higher incidence of cardiac arrhythmias and conductive defects has been reported in patients with PLSVC. This may be secondary to stretching and fibrosis of the atrioventricular node or His bundle by the dilated CS or associated sinus node dysfunction.^3^

Our patient was prone to infection or GI bleeding because of his underlying disorder and decreased immunocompetence. There was a risk of being manipulated with repeated IV catheter insertions and its mentioned complications. In addition, any dysrhythmia might be attributed to an underlying autoimmune disorder instead of an underlying neglected cardiac anomaly. Besides, the dilated CS has been known even to produce left ventricular inflow obstruction and heart failure.^21^

Since one of the most important medications used in AIP is corticosteroid therapy, which can lead to thromboembolic events itself, the risk of CVA in our patient could be estimated higher than usual. It is reasonable to exclude some of these rare disorders as Raghib syndrome, in cases that will initiate medications as corticosteroid therapies, which raise the probability of thromboembolic events. Lymphoplasmacytic aortitis has been previously described as an IgG4‐related disease.^22^ In our case, the aortic root was intact, and dimensions and elasticity had average values.

Most common congenital defects associated with combined great venous malformation included various types of atrial septal defects, endocardial cushion defects, and tetralogy of Fallot.^23^ In this case, the initial structural and functional disorders detected were mitral valve prolapse and mild mitral regurgitation, respectively.

The principal differential diagnosis for this finding would be indolent rheumatic mitral involvement, which could be differentiated from each other by WHO recommendations.^24^ According to these criteria, rheumatic involvement was excluded with a high probability.

Prednisolone was started for the patient, and he was a candidate for elective corrective surgery of unroofed LSVC. To the best of our knowledge, these two entities never have been reported to exist in the same patient. This infrequent association has not been reported yet, and there is no data that Raghib syndrome may have been the cause of autoimmunity. This issue must be followed up.

## 4 CONCLUSION

One of the most critical complications of PLSVC is paradoxical emboli. Medications as corticosteroids can lead to thromboembolic events themselves. It is reasonable to exclude some of these rare disorders as Raghib syndrome in cases who are going to be started on medications as corticosteroids which increases susceptibility to thromboembolic events. Particular attention should be paid to anatomical configuration such as dilated coronary sinus during echocardiography awareness about these conditions and comorbidities are essential to avoid complications.

## AUTHOR CONTRIBUTIONS

**Azizollah Yousefi:** Formal analysis. **Shima Salehi:** Writing – original draft; writing – review and editing. **Mohammad Radgoodarzi:** Formal analysis. **Asma Javid:** Writing – original draft.

## CONFLICT OF INTEREST STATEMENT

All the authors declare no conflict of interest.

## CONSENT

Written informed consent was obtained from the patient to publish this report in accordance with the journal's patient consent policy.

## Data Availability

All the data for used during the study are available from the patients archive of Rasool‐e‐ akram Hospital and after coordination with corresponding author.

## References

[1] Wreesmann V , van Eijck C , Naus D , van Velthuysen ML , Jeekel J , Mooi W . Inflammatory pseudotumor (inflammatory myofibroblastic tumor) of the pancreas: a report of six cases associated with obliterative phlebitis. Histopathology. 2001;38(2):105‐110. 11207823 10.1046/j.1365-2559.2001.01056.x

[2] Bolia R , Chong SY , Coleman L , MacGregor D , Hardikar W , Oliver MR . Autoimmune pancreatitis and IgG4 related disease in three children. ACG Case Rep J. 2016;3(4):113‐117. 10.14309/crj.2016.88 PMC5018227 27622194

[3] Zhang L , Smyrk TC . Autoimmune pancreatitis and IgG4‐related systemic diseases. Int J Clin Exp Pathol. 2010;3(5):491. 20606730 PMC2897107

[4] Kamisawa T , Funata N , Hayashi Y , et al. A new clinicopathological entity of IgG4‐related autoimmune disease. J Gastroenterol. 2003;38(10):982‐984. 14614606 10.1007/s00535-003-1175-y

[5] Zen Y , Grammatikopoulos T , Hadzic N . Autoimmune pancreatitis in children: insights into the diagnostic challenge. J Pediatr Gastroenterol Nutr. 2014;59(5):e42‐e45. 25347159 10.1097/MPG.0b013e3182994559

[6] Danielpour PJ , Aalberg JK , El‐Ramey M , Sivina M , Wodnicki H . Persistent left superior vena cava: an incidental finding during central venous catheterization: a case report. Vasc Endovasc Surg. 2005;39(1):109‐111. 10.1177/153857440503900111 15696254

[7] Chandra A , Reul GJ Jr . Persistent left superior vena cava. Discovered during placement of a central venous catheter. Tex Heart Inst J. 1998;25(1):90. 9566074 PMC325512

[8] Zellers TM , Hagler DJ , Julsrud PR . Accuracy of two‐dimensional echocardiography in diagnosing left superior vena cava. J Am Soc Echocardiogr. 1989;2(2):132‐138. 2629862 10.1016/s0894-7317(89)80076-8

[9] Konecky N , Freedberg RS , McCauley D , Kronzon I . Absent right and persistent left superior vena cava without other congenital anomalies: a rare combination diagnosed by transesophageal echocardiography. J Am Soc Echocardiogr. 1995;8(5):761‐766. 9417226 10.1016/s0894-7317(05)80397-9

[10] Soward A , ten Gate F , Fioretti P , Roelandt J , Serruys PW . An elusive persistent left superior vena cava draining into the left atrium. Cardiology. 1986;73(6):368‐371. 3791336 10.1159/000174030

[11] Long J , Birken G , Migicovsky B . Autoimmune pancreatitis in a child presenting as a pancreatic mass. J Pediatr Surg Case Rep. 2015;3(3):111‐113.

[12] Zamboni G , Lüttges J , Capelli P , et al. Histopathological features of diagnostic and clinical relevance in autoimmune pancreatitis: a study on 53 resection specimens and 9 biopsy specimens. Virchows Arch. 2004;445(6):552‐563. 15517359 10.1007/s00428-004-1140-z

[13] Hirano K , Kawabe T , Yamamoto N , et al. Serum IgG4 concentrations in pancreatic and biliary diseases. Clin Chim Acta. 2006;367(1‐2):181‐184. 16426597 10.1016/j.cca.2005.11.031

[14] Chari ST . Diagnosis of autoimmune pancreatitis using its five cardinal features: introducing the Mayo Clinic's HISORt criteria. J Gastroenterol. 2007;42(18):39‐41. 10.1007/s00535-007-2046-8 17520222

[15] Deheragoda MG , Church NI , Rodriguez–Justo M , et al. The use of immunoglobulin g4 immunostaining in diagnosing pancreatic and extrapancreatic involvement in autoimmune pancreatitis. Clin Gastroenterol Hepatol. 2007;5(10):1229‐1234. 17702660 10.1016/j.cgh.2007.04.023

[16] Fukukura Y , Fujiyoshi F , Nakamura F , Hamada H , Nakajo M . Autoimmune pancreatitis associated with idiopathic retroperitoneal fibrosis. Am J Roentgenol. 2003;181(4):993‐995. 14500215 10.2214/ajr.181.4.1810993

[17] Moorthy N , Kapoor A , Kumar S . Isolated persistent left‐sided superior vena cava, giant coronary sinus, atrial tachycardia, and heart failure in a child. Indian Heart J. 2013;65(5):603‐606. 24206885 10.1016/j.ihj.2013.08.024 PMC3860611

[18] Jacob M , Sokoll A , Mannherz HG . A case of persistent left and absent right superior caval vein: an anatomical and embryological perspective. Clin Anat. 2010;23(3):277‐286. 20169608 10.1002/ca.20945

[19] Kurtoglu E , Cakin O , Akcay S , Akturk E , Korkmaz H . Persistent left superior vena cava draining into the coronary sinus: a case report. Cardiol Res. 2011;2(5):249. 28357015 10.4021/cr85w PMC5358287

[20] Biffi M , Bertini M , Ziacchi M , et al. Clinical implications of left superior vena cava persistence in candidates for pacemaker or cardioverter‐defibrillator implantation. Heart Vessel. 2009;24(2):142‐146. 10.1007/s00380-008-1091-4 19337799

[21] DiBardino DJ , Fraser CD Jr , Dickerson HA , Heinle JS , McKenzie ED , Kung G . Left ventricular inflow obstruction associated with persistent left superior vena cava and dilated coronary sinus. J Thorac Cardiovasc Surg. 2004;127(4):959‐962. 15052190 10.1016/j.jtcvs.2003.07.010

[22] Ozawa M , Fujinaga Y , Asano J , et al. Clinical features of IgG4‐related periaortitis/periarteritis based on the analysis of 179 patients with IgG4‐related disease: a case‐control study. Arthritis Res Ther. 2017;19(1):223. 28978347 10.1186/s13075-017-1432-8 PMC5628426

[23] Bartram U , Van Praagh S , Levine JC , Hines M , Bensky AS , Van Praagh R . Absent right superior vena cava in visceroatrial situs solitus. Am J Cardiol. 1997;80(2):175‐183. 9230155 10.1016/s0002-9149(97)00314-7

[24] Vijayalakshmi IB , Mithravinda J , Deva ANP . The role of echocardiography in diagnosing carditis in the setting of acute rheumatic fever. Cardiol Young. 2005;15(6):583‐588. 16297251 10.1017/S1047951105001745

